# Sleep

**Published:** 1893-01

**Authors:** 


### SLEEP.

It is said by scientists to be a fact that all our senses do not slumber simultaneously, but that they fall into a happy state of insensibility one after another. Many dreams are explainable upon this hypothesis. The eyelids take the lead and obscure sight, the sense of taste is the next to lose its susceptibility, then follow smelling, hearing and touch; the last named being the lightest sleeper and most easily aroused. It is curious that, although the sense of smell is one of the first to slumber, it is the last to awake. Hearing, after touch, soonest regains consciousness. Certain muscles and parts of the body begin to sleep before others. Commencing with the feet, the slumberous influence works its way gradually upward to the centre of nervous action.

